# Surgery for non-Covid-19 patients during the pandemic

**DOI:** 10.1371/journal.pone.0241331

**Published:** 2020-10-23

**Authors:** Martin Hübner, Tobias Zingg, David Martin, Philippe Eckert, Nicolas Demartines

**Affiliations:** 1 Department of Visceral Surgery, University Hospital CHUV, University of Lausanne (UNIL), Lausanne, Switzerland; 2 Hospital direction, University Hospital CHUV, Lausanne, Switzerland; Technion - Israel Institute of Technology, ISRAEL

## Abstract

**Background:**

In the early phase of the Covid-19 pandemic, mainly data related to the burden of care required by infected patients were reported. The aim of this study was to illustrate the timeline of actions taken and to measure and analyze their impact on surgical patients.

**Method:**

This is a retrospective review of actions to limit Covid-19 spread and their impact on surgical activity in a Swiss tertiary referral center. Data on patient care, human resources and hospital logistics were collected. Impact on surgical activity was measured by comparing 6-week periods before and after the first measures were taken.

**Results:**

After the first Swiss Covid-19 case appeared on February 25, progressively restrictive measures were taken over a period of 23 days. Covid-19 positive inpatients increased from 5 to 131, and ICU patients from 2 to 31, between days 10 and 30, respectively, without ever overloading resources. A 43% decrease of elective visceral surgical procedures was observed after Covid-19 (295 vs 165, p<0.01), while the urgent operations (all specialties) decreased by 39% (1476 vs 897, p<0.01). Fifty-two and 38 major oncological surgeries were performed, respectively, representing a 27% decrease (p = 0.316). Outpatient consultations dropped by 59%, from 728 to 296 (p<0.01).

**Conclusion:**

While allowing for maximal care of Covid-19 patients during the pandemic, the shift of resources limited the access to elective surgical care, with less impact on cancer care.

## Introduction

The coronavirus (Covid-19) reached Europe on January 24, 2020 (first patient tested positive), while the first death was observed on February 14, 2020 [1]. On March 11, the WHO raised its status from epidemic to pandemic [2]. On May 3, the Swiss Federal Office of Public Health reported 29’905 confirmed Covid-19 cases causing 1473 (4.9%) fatalities [1]. According to data based on early reports from China, only 5−10% of symptomatic cases require inpatient treatment [3].

Most European countries imposed social distancing to decrease the number of new infections and reduce the load of inpatients, especially those requiring intensive care for ventilatory support. Hospitals had to anticipate and adapt to the new situation by increasing their surge capacities in their emergency, internal medicine and intensive care departments. A considerable shift of human and infrastructural resources was launched, at the cost of restraining surgical activity. Several countries prohibited non-essential elective and even oncological procedures for an indeterminate period of time [4–6].

Little was known on the impact of type, timing and duration of these measures, neither on the dynamics of the pandemic, nor on the care of non-Covid-19 patients. Scheduled operations for non-Covid-19 patients had to be postponed, for at least 2 months, based on Chinese data [3].

The aim of the present study was to describe the chronology of actions taken to mitigiate the effects of Covid-19 and to analyze their impact on surgical patients in a tertiary center.

## Materials and methods

This is a retrospective analysis of actions and their impact on coping with the Covid-19 crisis at Lausanne University Hospital (CHUV) in Western Switzerland. The latter is a tertiary center, with 1’554 beds and more than 10’400 employees. The Department of Visceral Surgery has an annual volume (2019) of 3318 interventions, including 1961 (59%) elective and 1357 (41%) urgent interventions.

The following actions were considered: (I) political decisions on national and regional (Vaud canton) level, (II) institutional decisions for the entire hospital, and (III) actions within the Department of Visceral Surgery. Actions at national and regional level were extracted on the basis of press releases from the Federal Office of Public Health (https://www.bag.admin.ch). Those of the hospital and the Department of Visceral Surgery were recovered from the reports of the weekly crisis meetings bringing together the Chiefdom of Departments. These data were accessed on April 30, 2020. A synopsis of all measures is presented on a common timeline starting at day 0, defined by the first reported Covid-19 patient in Switzerland on February 25, 2020.

Early outcomes of these actions aiming to contain virus spread and create additional resources were measured as follows: number of Covid-19 inpatients on wards, intensive care units and Covid-19 related deaths. ICU capacity was described by number of available and occupied ICU beds, by both Covid-19 positive and negative patients, and by available operating room (OR) capacities.

By comparing a six-week period from just before (February 03 –March 13) and during (March 16 –April 24) the pandemic, the impact on the Department of Visceral Surgery was assessed by number of elective visceral surgery operations (oncological and non-oncological), number of urgent operations (all specialties), number of outpatient consultations and by the need for modifications of oncological management strategies.

The numbers of tested and Covid-19 positives among the health care personnel were assessed. According to institutional guidelines, staff with symptoms (respiratory tract infections and/or fever and/or anosmia and/or ageusia) or asymptomatic staff having had close contact and living with a confirmed Covid-19 case should be screened (swab-PCR). Employees who were tested positive had to isolate themselves at home for 10 days and were able to come back to work after 48 hours without symptoms. Details of the demographics of each employee who was tested positively were not available (medical confidentiality). The sick leave rate for all hospital staff has been evaluated for the month of March 2020, and compared to March 2019.

Descriptive statistics for categorical variables were reported as frequencies and percentages, while continuous variables were reported as means (standard deviation) and compared with Mann–Whitney U test. A p value ≤ 0.05 was considered statistically significant. Analyses were performed using SPSS 25.0 software (SPSS Inc., Chicago, IL). All numbers were obtained from the hospital’s general direction using administrative databases containing no individual patient data. The local Committee for Medical and Health Research Ethics suggested that no formal ethical approval was required for this study.

## Results

**Fig 1:**
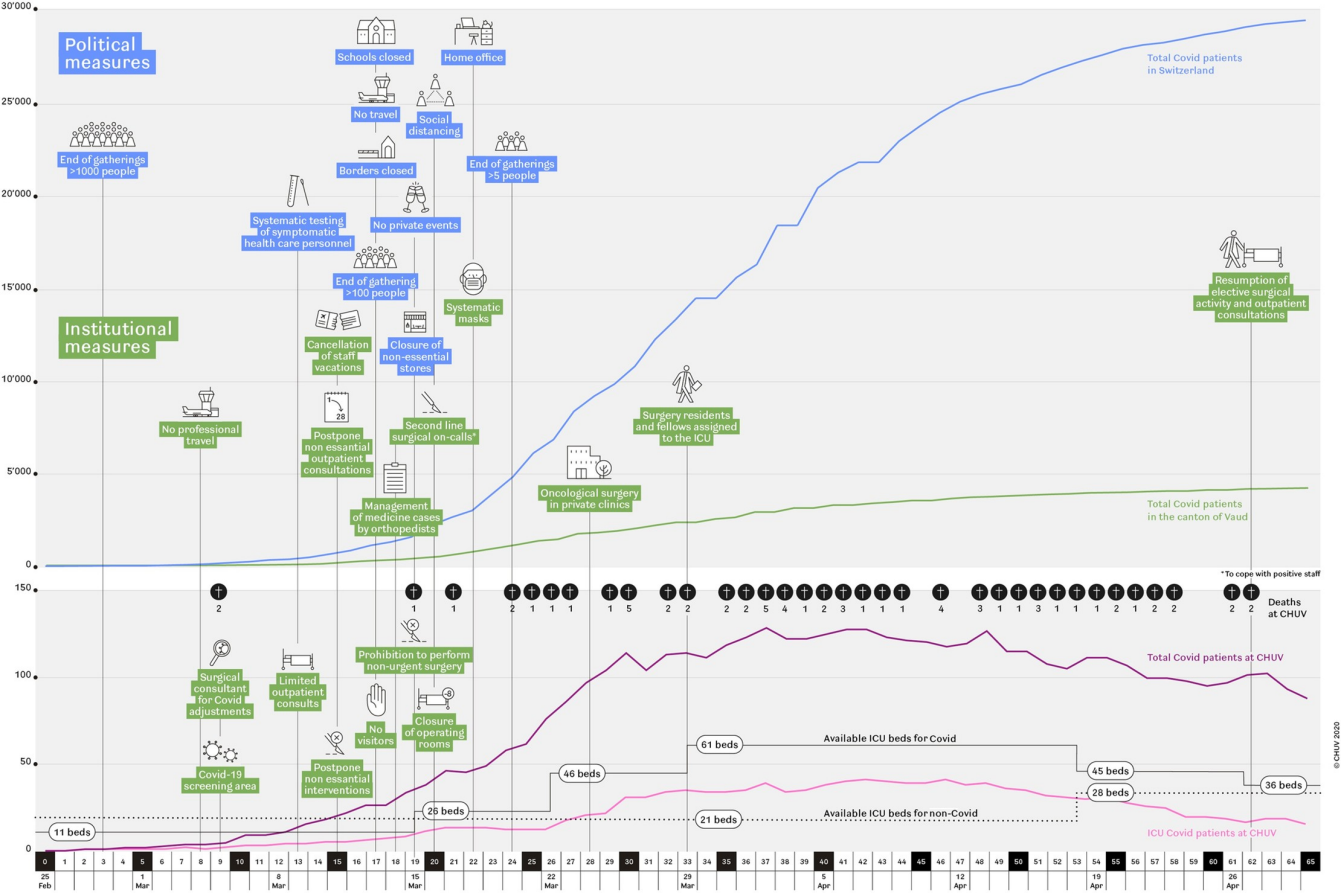
Synopsis of Covid-19 pandemic in University Hospital CHUV, Lausanne.

Political actions for confinement of the pandemic started on Day 3 and are summarized in **Fig 1**. Institutional measures were ordered on Day 8 and aimed at protecting collaborators and patients by cohorting, as well as increasing surge capacity in internal medicine, emergency and intensive care units.

The ICU capacity specific for Covid-19 patients was extended in a stepwise fashion from 11 at baseline to 26 (Day 19), 46 (Day 26) and up to 61 (Day 33) beds. To allow for this increase, 50% (8/16) of ORs were transformed into ICU suites, accommodating two ventilated patients each. A total of 21 “clean” ICU beds were kept for non-Covid-19 patients throughout the surge. Expansion to a theoretical capacity of 100 ICU beds could be achieved by including the recovery room, and thus further reducing the OR capacities. Transferring ICU patients to regional public and private hospitals allowed to offload two ICU beds on Day 26, and since then one additional Covid-19 ICU bed for several days.

On Day 34, 32 anesthesiologists and 10 surgeons were reassigned from the OR to staff the new ICUs. A total of 335 medical and nursing staff were assigned to work outside of their usual area, with 71 being shifted to the emergency department (ED) and the ICU. The number of health-care staff in the ICU increased from 250 before the pandemic to 700 at Day 34. During the same time, the sick-leave rate increased from an average of 350 hospital workers in March 2019 to 960 in March 2020 (+174%).

More than 15’000 Covid-19 naso-pharyneal swabs were analyzed by PCR since the beginning of the pandemic (in five daily batch runs). Test results were available within four hours after each run. A total number of 1793 tests were performed on symptomatic health-care personnel, of which 325 were positive (18.1%). The positive rate among the general population in our region during the same period was 20%. A single surgeon was tested positively in the Department of Visceral Surgery and quarantined for 10 days, which had no impact on surgical services.

A total of 295 elective visceral surgical procedures were performed before Covid-19 measures compared to 165 after Covid-19, representing a 43% decrease in activity, while the number of urgent operations decreased by 39% (**Table 1**). Specific description of the types of procedures provided during these periods are summarized in S1 Table. Significant decreases were observed for cholecystectomies (10.5 Vs. 5.5%, p = 0.065), bariatric surgery (3.7 Vs. 0%, p = 0.012) and proctological procedures 13.6% Vs 0%, p<0.01). Overall operating time decreased by respectively 40% and 39% for elective and urgent operations over this same period. The number of outpatient consultations dropped by 59%, from 728 to 296.

**Table 1:** Covid-19 impact on a department of visceral surgery.

	6 weeks	6 weeks	Variation	P value *
	pre Covid-19	post Covid-19	%	
**Elective visceral surgery**				
Operations, n	295	165	43	**< 0.01**
Oncological operations, n	52	38	27	0.316
Daily operations, mean (SD)	7 (3)	4 (2)	43	**< 0.01**
**Urgent surgery (all specialties)**				
Operations, n	1476	897	39	**< 0.01**
Daily operations, mean (SD)	36 (12)	22 (7)	39	**< 0.01**
**Outpatient**				
Consultations, n	728	296	59	**< 0.01**
Daily consultations, mean (SD)	24 (21)	10 (9)	58	**< 0.01**

* Comparison of pre- and post-Covid-19 pandemic.

Significant p values (<0.01) are displayed in bold characters.

SD: standard deviation.

During the two phases, 52 and 38 major oncological surgeries were performed, respectively, representing a 27% decrease due to Covid-19 measures (p = 0.316). In collaboration with medical oncology services, adaptive modifications of current treatment standards with valid alternatives were elaborated for several clinical scenarios (S2 Table). Modifications of current treatment standards were considered inacceptable when no valid alternative treatment was available or when the potential alternative was likely to worsen the oncological outcome. Examples included curative resection of T2/3 N0 colon cancer, upfront surgery of resectable low risk cancer hepato-pancreato-biliary tumours, and cytoreduction and HIPEC in pseudomyxoma.

By May 1^st^, 467 cumulative Covid-19 patients were hospitalized, of whom 273 were discharged home, 126 were transferred to acute care or rehabilitation hospitals, and 68 died. The peak of inpatient (149) and intensive care patients (40) were encountered on days 37 and 42, respectively, and the cases started to decrease. Due to similar results in other institutions, a 3-step exit strategy over 4 weeks on national level and re-start of elective surgery and outpatients consultations was launched on April 27. The duration of this first phase lasted hence about 6 weeks.

It was too early to measure the real financial impact of the pandemic on the institution, as staff cost and loss of revenue due to decreased consultations and operations are currently unknown. The range will depend on the time of normal activity resumption.

## Discussion

The Covid-19 pandemic made a shift of resources necessary and significantly limited the access to elective surgical care, with less impact on cancer care.

In terms of impact on daily hospital activity, the dynamics with increasing Covid-19 cases from one inpatient on Day 6 to 149 by Day 37 called for adaptation of infrastructural and human resources with a shift in priorities having a measurable impact on surgical patient care. **Fig 1** shows the timeline of exponential increase in both case numbers and ICU capacity, as well as the measures taken. Switzerland was fortunate to have observations from China (January 2020) and Italy (February 2020), therefore able to anticipate the changes induced by the pandemic on national health systems.

**Fig 2:**
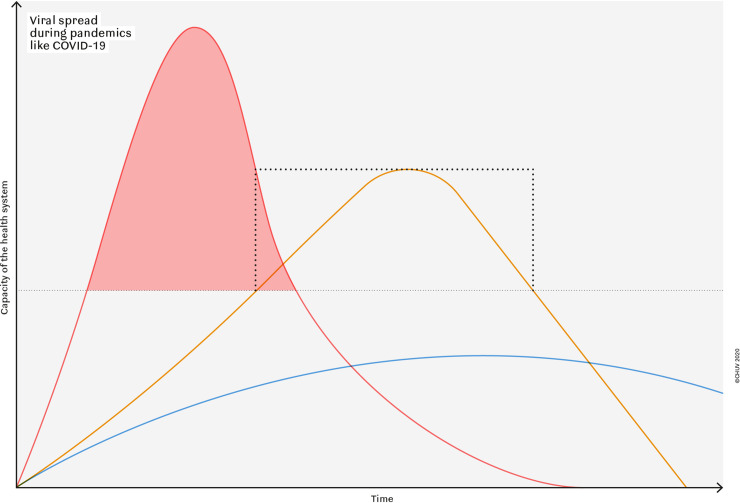
Viral spread during pandemics like Covid-19. Coping measures during pandemics include increase / shifting of resources to make hospitals more performant and to contain viral spread to slow down dynamics and to flatten the peak. The ideal defence (middle) entails optimal choice and onset of actions to provide enough resources for acute patient care (no overcharge of system: red area). Containment measures and shift of resources should be restricted to the minimal duration due to their inevitable deleterious effects on care of other patients, economy and sociocultural life.

“To flatten the curve” of hospitalization aims at avoiding a patients/resources mismatch (**Fig 2**), and at gaining time to create more resources and increasing surge capacity. Of note, intubation and mechanical ventilation for Covid-19 patients generally lasts between 10 and 15 days [7]. Therefore, hospitalized patients stay up to 3 to 4 weeks in the hospital. For this reason, it’s important to slow down the rate of contagion and avoid a peak overwhelming health-care services [7], which, due to the taken measures, could be avoided in the case of our institution. Indeed, a flattening of the epidemiological curve (**Fig 1**) has been observed in a relatively short period of time, thus confirming a certain effectiveness of the measures taken. The duration of this first phase lasted about 6 weeks also justifying analysis and reporting of this timeframe which could serve as a "template" for possible future similar pandemics.

True infection rates however remains unknown and initial data suggested that about 80% of people with Covid-19 were either asymptomatic or had a mild course, 14% presented with severe disease, and 6% critical illness [7]. Crude mortality in the present study seemed comparable to other countries [7, 8].

The required measures were shifts of manpower, hospital beds and ICU capacities by transformation of surgical areas into ICUs. Human resources were reallocated to the ED and ICUs from other areas, including surgery and anaesthesia. The ICU capacity dedicated to Covid-19 increased from initially 11 to a targeted maximal number of 100 ventilator beds in several steps (+900%) during the pandemic. In addition, adaptations were required, such as homeoffice work, video conference and telephone consultation. Several studies have reported interesting perspectives of telemedicine in the surgical context, especially due to economic benefit, reduced missed work and reduced trips to hospital [9–11]. A survey on public’s perception of telemedicine showed that it was an acceptable substitute for in-person visits, especially during the pandemic [10]. It must be mentioned too that some providers worked in disciplines that were not their areas and were probably less comfortable in. Their effectiveness as well as their possible impact on the quality of care are difficult to assess and are currently unknown.

Another important factor is the collaboration with both public and private regional hospitals, who also increased their ICU capacities in order to relieve our tertiary center to accommodate the most serious cases. Furthermore, criteria for limiting admission to ICU have been established on the basis of a consensus between ICU consultants by considering the evidence in the literature and their clinical experience (cardio-respiratory arrest without witness or return of spontaneous circulation, oncological disease with life expectancy of less than 1 year, end-stage neurodegenerative disease, severe dementia, irreversible central neurological damage, NYHA IV heart failure, cirrhosis with CHILD score > 8). In addition, patients with Covid-19 remained in their nursing home unless they had a diagnosis requiring immediate surgery. Moreover, in the same period, patients who recovered from Covid-19 started to return home. These parameters have probably contributed to flatten the curve of hospitalisation.

Concerning impact on non-Covid-19 patients, as described for other outbreaks such as Ebola, mortality from other causes increased as a consequence of a saturated health system and disease or deaths among health-care workers [12]. While disease of health care workers seems of limited impact for now, despite the pandemic, non-Covid-19 patients, especially oncological, still deserve high quality management. On the other hand, with the pandemic expanding, further limitation of resources may be expected. Therefore, alternatives to surgery needed to be anticipated. **S1 Table** provides an overview of management modifications for surgical oncology patients during the Covid-19 pandemic, in response to the reduced OR and anaesthesia capacities. This adapted strategy was established within our institutional tumour board, based only on scientificly validated and ethical alternatives to immediate surgery. However, the Covid-19 pandemic currently significantly limited the access to elective visceral surgical care (43% decrease), with less impact on cancer care (27% decrease). Another retrospective study showed higher rates of decrease in surgical activity, between 80 and 90% depending on the specialty [13]. Using data from hospitals across 190 countries, CovidSurg Collaborative researchers created a statistical model to estimate the total number of canceled elective surgeries during 12 weeks of peak disruption [14]. According to their estimates, 28’404’603 operations would be cancelled or postponed, concerning 81.7% of benign surgery and 37.7% of cancer surgery [14]. Delaying cancer may lead to deteriorating health, worsening quality of life, and unnecessary deaths [14, 15]. On the contrary, in the present study, urgent interventions have always been provided, 24 hours a day, all specialties combined, as usual before the pandemic. No operating room was closed, but interestingly, emergency surgical activity significantly decreased during this same period (39% decrease). Another retrospective review also showed that total surgical consult volume decreased by 43% in the post-COVID-19 period, with most operation types decreasing in frequency [16]. One of the hypotheses for this phenomenon is the higher threshold for presentation to the emergency department, by either self-referral or physician-referral, due to stay-at-home orders and fear of Covid-19 exposure in the hospital itself [16].

Temporary shifting of resource allocation may be acceptable for a limited period of time, but serious medical, ethical and organizational issues will need to be handled sooner or later. Determining the temporal extent of all taken measures will therefore be a key factor in the months to come based on the evolution of the epidemic. Similarly, the financial impact remains currently unknown. The loss will be due to additional costs and loss of revenue. The loss of revenue is due to the suspension of elective activities (outpatient consultations, interventions, hospitalizations) from mid-March to the end of April. The additional costs are due to all measures to fight the epidemic (additional staff and ICU beds, purchase of personal protective equipement).

One way to cope with the Covid-19 pandemic entails resources and strategies to absorb the surge in Covid-19 patients to be treated. At the same time, it is of importance to maintain equipoise for non-Covid-19 patients despite decreased resources. Adaptations of surgical guidelines are required, as recommended by various scientific societies [6, 17, 18]. Thus, the “*primum non nocere*” principle has to be applied not only for Covid-19, but all patients. The global society is interconnected and politicians and health care managers should learn from Covid-19 management [3]. The good and bad experiences in different countries and health systems should be discussed within the authorities, in particular by maintaining strong links between the scientific and political communities in order to fight against the spread of the virus. International surveillance, cooperation, coordination, and communication are required. A Bayesian regression model showed that if countries increased their normal surgical volume by 20 per cent after the pandemic, it would take a median of 45 weeks to clear the backlog of operations resulting from COVID-19 disruption [14].

The present analysis is descriptive and reports only the early evolution of the Covid-19 pandemic from the perspective of one tertiary referral centre in Western Switzerland. Short-term patient outcomes, such as the intensive care stay, the duration of intubation, morbidity, sequelae, overall length of stay and transfers for example, were not available. These data rather concern the Covid pathology per se, and not the actions taken for the crisis, which was the aim of the study. Moreover, reporting long-term outcomes was neither the scope nor the purpose of the present paper. Health care personnel tested positive for Covid-19 could not be coupled with loss of human resources and how this impacted on providing surgical services (medical confidentiality). Furthermore, data on Covid-19 patients on ward and surgical patients (ward and ICU) during these periods were not available, as were the data on emergencies (all specialties). Likewise, details of surgical consultations (maintenance, cancellation, telemedicine) were not collected. Comparisons or extrapolations to other institutions and countries are therefore problematic. Nevertheless, it appears important to report data on early experience now, while many countries are preparing for the surge, or in case of similar pandemics in the future.

## Conclusions

Unusual, early and vigorous actions and their progressive and timely implementation have allowed to attenuate the Covid-19 crisis by shifting resources. A significant decrease in surgical activity was measured, with less impact on major oncologic operations. The consequences on public health, health costs and surgical oncology outcome need to be monitored and assessed by reporting not only the COVID-19 pandemic consequences but also its collateral effects on non-Covid-19 patients.

## Supporting information

S1 Table Surgical procedures before and after onset of the COVID-19 pandemic. (DOCX)

S2 Table Adaptation of oncological surgery in the times of COVID-19 pandemic. (DOCX)
