# Supplementary material for: Surgery for non-Covid-19 patients during the pandemic
Source: PLoS One. 2020 Oct 23;15(10):e0241331. doi: 10.1371/journal.pone.0241331 (PMC7584248; doi:10.1371/journal.pone.0241331)
Supplement: S1 Table — (DOCX) [file pone.0241331.s001.docx]

**S1 Table** Surgical procedures before and after onset of the COVID-19 pandemic

|  | **6 weeks BEFORE**  **(n=295)** | **6 weeks AFTER**  **(n=165)** | **P value *** |
| --- | --- | --- | --- |
| **Eventration** | 15 (5.1%) | 3 (1.8%) | 0.083 |
| **Primary hernia** | 19 (6.4%) | 7 (4.2%) | 0.327 |
| **Cholecystectomy** | 31 (10.5%) | 9 (5.5%) | **0.065** |
| **Liver, bile ducts** | 12 (4.1%) | 13 (7.9%) | 0.084 |
| **Pancreas** | 9 (3.0%) | 13 (7.9%) | **0.020** |
| **Stomach, duodenum** | 5 (1.7%) | 5 (3.0%) | 0.346 |
| **Fundoplication** | 9 (3.1%) | 5 (3.0%) | 0.990 |
| **Esophagus** | 4 (1.4%) | 4 (2.4%) | 0.401 |
| **Bariatric surgery** | 11 (3.7%) | 0 (-) | **0.012** |
| **Colorectal (other)** | 21 (7.1%) | 17 (10.4%) | 0.234 |
| **Colon** | 17 (5.8%) | 10 (6.0%) | 0.896 |
| **Rectum** | 8 (2.7%) | 6 (3.6%) | 0.580 |
| **Ileostomy closure** | 7 (2.4%) | 4 (2.4%) | 0.972 |
| **Proctology** | 40 (13.6%) | 0 (-) | **< 0.01** |
| **PIPAC*** | 14 (4.7%) | 6 (3.6%) | 0.642 |
| **Nephrectomy** | 5 (1.7%) | 4 (2.4%) | 0.923 |
| **Kidney transplantation** | 1 (0.4%) | 0 (-) | 0.454 |
| **Melanoma, lymph nodes** | 12 (4.1%) | 10 (6.0%) | 0.337 |
| **Endocrine** | 8 (2.7%) | 6 (3.6%) | 0.582 |
| **Implanted vascular access device** | 24 (8.1%) | 26 (15.9%) | **0.012** |
| **Other** | 23 (7.7%) | 17 (10.4%) | 0.483 |

* Significant p values (<0.05) are displayed in bold characters.

PIPAC: Pressurized Intra Peritoneal Aerosol Chemotherapy
