# Supplementary material for: Surgery for non-Covid-19 patients during the pandemic
Source: PLoS One. 2020 Oct 23;15(10):e0241331. doi: 10.1371/journal.pone.0241331 (PMC7584248; doi:10.1371/journal.pone.0241331)
Supplement: S2 Table — (DOCX) [file pone.0241331.s002.docx]

**S2 Table** Adaptation of oncological surgery in the times of COVID-19 pandemic

A Extended time interval

| Tumor entity | Clinical setting | Standard care | Modification |
| --- | --- | --- | --- |
| Colon | cT2/T3 N0 | Surgery <2 weeks | Surgery <6 weeks [^15^](#_ENREF_15) |
| Colorectal tumor and liver metastases | Resectable | Chemotherapy (3months) – liver resection – chemotherapy – resection of primary | Chemotherapy (6 months) – re-staging |

B Altered treatment sequence

| Tumor entity | Clinical setting | Standard care | Modification |
| --- | --- | --- | --- |
| Pancreas | Localised | Surgery – adjuvant chemotherapy | Neoadjuvant chemotherapy – re-staging – surgery |
| Colon | cT3/T4 or N+ | Surgery – adjuvant chemotherapy | Neoadjuvant chemotherapy (FOxTROT[^16^](#_ENREF_16) and PRODIGE 22[^17^](#_ENREF_17) trials) – Surgery at (6-)12 weeks |
| Esophagus | Adenocarcinoma, T3-4 N+ | 4x FLOT – surgery – 4x FLOT | 8x FLOT – re-staging – surgery |
| Stomach | T3-4 N+ | 4x FLOT – surgery – 4x FLOT | 8x FLOT – re-staging – surgery |

C Change in treatment or treatment strategy

| Tumor entity | Clinical setting | Standard care | Modification |
| --- | --- | --- | --- |
| Hepatocellular carcinoma | Resectable | Surgery | SIRT |
| Gallbladder cancer | cT4, resectable | Surgery | Neoadjuvant chemotherapy – re-staging |
| Rectum | mrT2, 3a, clear CRM, non EMVI | Surgery | RT 5x5Gy – surgery at 12 weeks |
|  | ≥mrT3b or compromised CRM or EMVI | CRT – Surgery | RT 5x5Gy – surgery at 12 weeks |
| Colon | Cancerous polyp (sm 2,3) | Surgery | Close follow-up (CT, endoscopy) |
| Mesothelioma | Resectable | CRS, HIPEC | Neoadjuvant chemotherapy (3x Cisplatin, Alimta) – CRS+HIPEC |
| Stomach | T2 N0 | Surgery | Neoadjuvant chemotherapy (4x FLOT) - surgery |
| Esophagus | Squamous cell,  T1b-T2 N0 | Surgery | CRT (50.4 Gy) – re-staging at 12 weeks – surgery (if presence of tumor) |
|  | Adenocarcinoma,  T1b-T2 N0 | Surgery | 4x FLOT – surgery – 4x FLOT  Or  8x FLOT - Surgery |

Legend

FLOT: Docetaxel, oxaliplatin, leucovorin, and 5-fluorouracil chemotherapy; SIRT: selective internal radiation therapy; CRM: Circumferential resection margin; EMVI: extramural venous invasion; CRT: chemoradiotherapy; RT: radiotherapy; CRS: cytoreduction chemotherapy; HIPEC: hyperthermic intraperitoneal chemotherapy.
